# A Standardized Classification of Uveal Injury

**DOI:** 10.1155/joph/8878849

**Published:** 2026-07-13

**Authors:** Tianjing Yang, Hua Yan

**Affiliations:** ^1^ School of Medicine, Nankai University, Tianjin, China, nankai.edu.cn; ^2^ Department of Ophthalmology, Tianjin Medical University General Hospital, Tianjin, China, tjmugh.com.cn; ^3^ Ministry of Education International Joint Laboratory of Ocular Diseases, Tianjin Medical University General Hospital, Tianjin, China, tjmugh.com.cn; ^4^ Tianjin Key Laboratory of Ocular Trauma, Tianjin Medical University General Hospital, Tianjin, China, tjmugh.com.cn; ^5^ Tianjin Institute of Eye Health and Eye Diseases, Tianjin Medical University General Hospital, Tianjin, China, tjmugh.com.cn; ^6^ Laboratory of Molecular Ophthalmology, Tianjin Medical University General Hospital, Tianjin, China, tjmugh.com.cn

**Keywords:** anatomical restoration, classification, open globe injury, uveal injury, vitrectomy

## Abstract

**Purpose:**

Mechanical ocular injury involving the uvea poses significant surgical challenges and risks of irreversible visual impairment. Currently, there is no standardized classification to guide the management and prognosis of uveal injuries. This study aims to establish such a classification system.

**Methods:**

A methodological proposal for a novel classification was developed based on the most posterior location of uveal laceration/rupture and the extent of quadrant involvement. The classification was informed by clinical data with uveal injury. The system is designed to be determinable during clinical examination, emergency repair, or vitrectomy.

**Results:**

This study proposes a novel, standardized classification system for uveal injury based on the most posterior location of uveal laceration or rupture: Zone I (iris), Zone II (ciliary body), Zone III (choroid anterior to the equator), and Zone IV (choroid posterior to the equator), each subdivided (a–d) by extent of quadrant involvement. The system defines reproducible surgical approaches per zone.

**Conclusion:**

This novel classification is presented as a proposal to standardize the assessment of uveal injury, addressing a critical gap in existing ocular trauma systems. It provides a structured framework to guide surgical decision‐making and anatomical restoration and to facilitate future research in the management of severe ocular trauma.

Mechanical ocular injury can cause severe ocular mobility and irreversible visual impairment [1]. The severity of structural damage varies depending on the type of trauma, encompassing entities such as traumatic cataract, ciliary body injury, vitreous hemorrhage, and retinal‐choroidal laceration, detachment, or even loss [2–4]. Recently, the ongoing advancement of vitreoretinal surgical techniques has markedly improved the success rate of trauma management [1, 5]. However, management of uveal injury due to severe ocular trauma remains challenging [6], regarding how to achieve long‐term anatomical restoration of the uvea through surgery with lower incidence of severe postoperative complications. Uveal injuries in different locations require correspondingly distinct surgical approaches. So far, there is no clearly defined classification for uveal injury. It is imperative to establish a detailed classification of uveal injury to guide the therapeutic approaches and evaluated prognosis in cases of severe ocular trauma with uvea involved.

## 1. Methods for Classification

From 2010 to 2025, a total of 108 patients (110 eyes) with uveal injury were diagnosed in the Department of Ophthalmology, Tianjin Medical University General Hospital. Among them, there were 38 cases (38 eyes) of iris root avulsion, 40 cases (40 eyes) of cyclodialysis, 18 cases (18 eyes) of choroidal injury (anterior to the ocular equator), and 12 cases (14 eyes) of choroidal injury (posterior to the ocular equator). Patients with iris root avulsion, cyclodialysis, and choroidal injury (anterior to the ocular equator) underwent suturing. For choroidal injury (posterior to the ocular equator), no suturing was performed; the detached choroid was repositioned under perfluorocarbon liquid, followed by silicone oil tamponade. Pars plana vitrectomy (PPV) surgery was completed within 7 days after injury. The final reposition rate was 100% for iris root avulsion and cyclodialysis, 83.3% for choroidal injury (anterior to the equator), and 92.9% for choroidal injury (posterior to the equator). Supporting Table S1 summarizes the demographic and injury characteristics of the 108 patients, including age, sex, injury mechanism, follow‐up duration, and complications.

**FIGURE 1:**
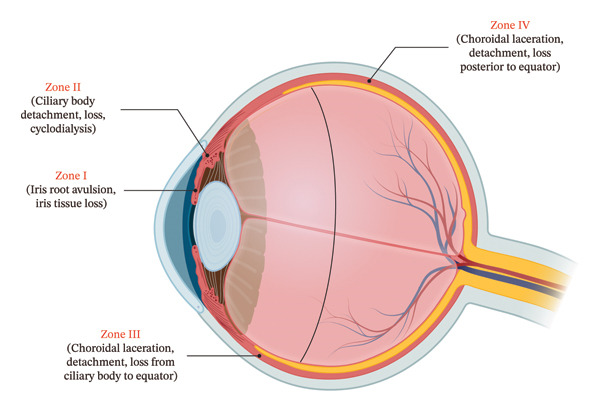
Schematic illustration of the four zones of uveal injury.

Based on the clinical data, we developed the following classification. Uveal injury is classified based on the most posterior location of the uveal laceration or rupture. Zone I injury refers to uveal injury confined to the iris, including iris root avulsion or iris tissue injury. Based on the extent of quadrants involved, it is subdivided into Ia, Ib, Ic, and Id. Zone II injury extends posteriorly to the ciliary body region, which is subdivided into IIa, IIb, IIc, and IId according to the extent of quadrants involved. Zone III injury refers to uveal injury involving the choroid anterior to the ocular equator, which is subdivided into IIIa, IIIb, IIIc, and IIId depending on the extent of involvement. Zone IV injury refers to uveal injury involving the choroid posterior to the ocular equator. Based on the extent of involvement, Zone IV injury is subdivided into IVa, IVb, IVc, and IVd (Table 1 and Figure 1).

**TABLE 1:** Classification of uveal injury.

Zone	Area of involvement	Representative injury patterns	Typical surgical considerations	Anticipated clinical challenges
I				
Ia	Involvement of 1 quadrant	Iris root avulsion, iris tissue loss	Anterior segment suture via scleral tunnel, direct suturing of avulsed iris	Traumatic mydriasis, photophobia, glare, cosmetic pupil abnormalities
Ib	Involvement of 2 quadrants			
Ic	Involvement of 3 quadrants			
Id	Involvement of 4 quadrants			
II				
IIa	Involvement of 1 quadrant	Ciliary body detachment, loss, cyclodialysis	Observation or laser cyclopexy for mild cases, direct suturing, endoscopic cyclopexy, or cryotherapy	Hypotony, hypotony maculopathy, dysfunction of accommodation
IIb	Involvement of 2 quadrants			
IIc	Involvement of 3 quadrants			
IId	Involvement of 4 quadrants			
III				
IIIa	Involvement of 1 quadrant	Choroidal laceration, detachment, loss from ciliary body to equator	Transscleral choroidal suturing, concurrent retinopexy or endolaser, vitrectomy, silicone oil, or gas tamponade	Proliferative vitreoretinopathy, retinal detachment, recurrent choroidal detachment
IIIb	Involvement of 2 quadrants			
IIIc	Involvement of 3 quadrants			
IIId	Involvement of 4 quadrants			
IV				
IVa	Involvement of 1 quadrant	Choroidal laceration, detachment, loss posterior to equator	No direct suturing, flatten residual choroid, retinal photocoagulation, vitrectomy, silicone oil tamponade	Proliferative vitreoretinopathy, fibrous adhesions, recurrent choroidal detachment, phthisis bulbi
IVb	Involvement of 2 quadrants			
IVc	Involvement of 3 quadrants			
IVd	Involvement of 4 quadrants			

The zone of uveal injury can be determined by professional ophthalmologists during the initial examination, or following emergency surgical treatment assistant with advanced ophthalmic equipment, or during emergency surgery and vitrectomy.

## 2. Surgical Approaches

### 2.1. Zone I Injury

In the corresponding area of iris root avulsion, create a lamellar scleral tunnel 1 mm posterior to the limbus. The avulsed iris is sutured directly for fixation.

### 2.2. Zone II Injury

The management of ciliary body injury depends on the severity and extent. For mild, nonavulsed cyclodialysis, observation or laser cyclopexy may be considered. For more extensive ciliary body detachment or avulsion, surgical intervention is indicated. In the corresponding area of ciliary body avulsion, a lamellar scleral tunnel is created 2 mm posterior to the limbus. The avulsed ciliary body is then sutured directly onto the sclera for fixation. Alternative techniques, such as endoscopic cyclopexy or cryotherapy, may also be employed depending on the clinical condition.

### 2.3. Zone III Injury

At the corresponding site of choroidal laceration or detachment, the needle is entered from the sclera and pass through the anterior edge of the lacerated or detached choroid and into the vitreous cavity. Simultaneously, on the scleral surface at the location of the opposite pars plana, a 1‐mL syringe needle is used to relay the suture needle from the vitreous cavity and pull it out. Then, re‐enter the suture needle from the original 1‐mL site, pass it through the lacerated or detached choroid, and pull it out through the scleral surface for tying. In principle, two sutures per quadrant are sufficient. In addition to choroidal management, concurrent retinal injuries such as retinal breaks or detachment should be addressed. Retinopexy or endolaser photocoagulation is applied around retinal lesions. If significant retinal detachment or traction is present, silicone oil or gas tamponade may be used after vitrectomy.

### 2.4. Zone IV Injury

Because the choroidal injury site is posterior to the equator, direct suture is unfeasible due to the influence of eye position. Therefore, the avulsed or detached choroid does not require suturing. After thoroughly treating the lesion, the residual choroid can be flattened directly under air‐fluid exchange. Subsequently, retinal photocoagulation therapy is performed followed by silicone oil tamponade.

## 3. Discussion

Mechanical ocular trauma can cause severe damage to ocular structures, including iris root avulsion, cyclodialysis, choroidal laceration or loss, retinal detachment, intraocular foreign body, and endophthalmitis [7]. Ferenc Kuhn [8] classified mechanical ocular trauma, and Pieramici [9] zoned ocular trauma, aiming to refine the classification of different ocular injuries from complex trauma to better guide clinical treatment. For decades, experience on managements of a large numbers of mechanical ocular trauma cases has revealed that existing classification lack sufficient details, particularly regarding intraocular tissue damage such as uveal injury [10]. Hence, the inability to classify for subsequent treatment guidance has hindered research and development in the management of choroidal injuries to some extent [6]. It is important to emphasize that the proposed uveal injury classification is not intended to replace existing ocular trauma classifications but rather to supplement them by providing a detailed categorization of uveal involvement, which has been lacking. The existing systems classify the globe injury and wound zones, while our system adds a uveal‐specific layer. A comparison of the strengths and limitations of each system is provided in Table S2.

We propose a novel classification system to assess both the location and extent of uveal damage due to mechanical trauma. The information provided by this classification system has quantifiable and practical characteristics for the diagnosis and treatment of uveal injuries. However, because uveal injury often occurs in severe ocular trauma, i.e., combined with corneal injury, hyphema, traumatic cataract, massive vitreous hemorrhage, and retinal detachment, it is difficult to determine the classification of uveal injury through routine ophthalmic examinations and imaging, which often requires assessment during vitrectomy surgery [6].

Regarding the treatment of uveal injury, management of Zone I or II uveal injury is relatively direct and easier to perform. There are various treatment methods, and suturing generally achieves uveal reposition. The difficulty in treating uveal injury lies in Zones III and IV when choroidal injuries are involved, especially with significantly lower anatomical repositioning rate [11, 12]. Of note, the choroid contains abundant elastic and collagen fibers, which indicates that once the choroidal tissue is ruptured, the elastic fibers will retract, thereby causing the avulsed choroid to shorten and difficult to reattach to the sclera. Choroidal avulsion and rupture create connection between the suprachoroidal space and the vitreous cavity. After choroidal detachment, the space of the vitreous cavity decreases, resulting in less operating space left during vitrectomy and increasing surgical difficulty. If the choroid cannot be anatomically repositioned, the retina loses its foundation for reattachment. Consequently, the residual retina fails to achieve anatomical reattachment, ultimately leading to surgical failure and vision loss [13]. Smaller choroidal ruptures can be sealed by cryotherapy or scleral buckling combined with silicone oil tamponade. Larger choroidal avulsion or detachment can be managed with transscleral choroidal suture [14, 15]. Clinical observation suggests that the mechanism of choroidal–scleral junction postsurgery primarily relies on tissue adhesion caused by local inflammatory reactions. Intraoperative choroidal suture only serves to temporarily fix the tissue. However, in cases with large choroidal rupture or loss located at the equator where choroidal suture is unfeasible, choroidal reposition under silicone oil tamponade also relies on local inflammation rather than mechanical flattening.

For Zone IV injuries where direct choroidal suturing is not feasible, PPV plays a central role in reattaching the avulsed or detached choroid and restoring intraocular architecture. The timing of vitrectomy is critical. The optimal timing should be identified during the dynamic pathophysiological process of intraocular trauma repair for the PPV treatment in terms of achieving the optimal prognosis [16–18]. Based on the current evidence and clinical experience, early vitrectomy performed within 0–7 days after injury is recommended for Zone IV injuries [19]. In particular, for posterior choroidal detachment or avulsion, early intervention can prevent the formation of fibrous adhesions and reduce the formation of proliferative vitreoretinopathy.

Open globe injuries account for the majority of uveal trauma in this series, with globe rupture being the most common subtype. These open injuries frequently lead to tissue prolapse, retinal–choroidal detachment, and suprachoroidal hemorrhage. In contrast, closed globe injuries, though less frequent, can also cause uveal damage such as hemorrhagic choroidal detachment, cyclodialysis, and iris root avulsion, albeit generally with less severity than open injuries. Severe ocular trauma involving the uvea is often accompanied by other injuries that significantly affect surgical complexity and visual prognosis. Retinal detachment and vitreous hemorrhage are most common in Zone III and IV injuries and typically require PPV with endolaser or cryotherapy, followed by silicone oil or gas tamponade after uveal repositioning. Traumatic cataract or lens dislocation may occur in any zone, especially when the ciliary body is involved, and lens extraction should be performed during the same surgery if it obstructs the view or when the lens is dislocated into the vitreous cavity. Globe rupture with uveal prolapse requires primary repair combined with uveal repositioning according to the involved zone. Intraocular foreign bodies should be removed via the most appropriate port (pars plana for posterior segment). Endophthalmitis, when suspected, takes priority; emergency vitrectomy and antibiotic injection are indicated before definitive uveal repair. Traumatic optic neuropathy may limit visual recovery regardless of successful anatomical restoration. The uveal zone classification guides the surgical approach for uveal injury but must be applied alongside appropriate management of these associated conditions.

The functional consequences of uveal injury also support the clinical relevance of the proposed zonal classification. Iris injuries (Zone I) may result in traumatic mydriasis, photophobia, glare, and cosmetic pupil abnormalities; these can be managed with pupil‐forming procedures or artificial iris implantation when symptomatic [20]. Ciliary body injuries (Zone II) can lead to hypotony, hypotony maculopathy, and dysfunction of accommodation; surgical cyclopexy or laser coagulation may restore intraocular pressure, and medical therapy (e.g., cycloplegics) can help control symptoms. Choroidal injuries (Zones III and IV) may cause retinal dysfunction, choroidal neovascularization, and proliferative vitreoretinopathy; management includes anti‐VEGF injections for neovascularization, early vitrectomy for proliferative vitreoretinopathy, and close monitoring of retinal function. These distinct functional outcomes, which often correlate with the anatomical zone of injury, underscore the importance of accurately identifying the most posterior extent of uveal damage. The proposed classification provides a structured framework for anticipating these complications and planning appropriate surgical and medical management.

However, this study has certain limitations. The proposed classification is currently based on retrospective clinical data, anatomical principles, and surgical experience but lacks prospective, multicenter validation. These data are provided for illustrative purposes only to support the classification, not as a complete outcomes study. Future studies are required to validate whether this classification system and its treatment decisions can lead to better surgical outcomes and visual prognosis. The surgical approaches for each zone are based on the authors’ institutional experience and are offered as suggested management options. Individual cases may require modification depending on specific injury characteristics and surgeon judgment.

The system is primarily designed as an anatomical and surgical classification to guide surgical decision‐making by identifying the most posterior extent of uveal injury and the number of quadrants involved. Although more posterior uveal involvement (Zones III and IV) and greater quadrant extent (Subgroups c and d) are hypothesized to correlate with worse visual outcomes due to a higher likelihood of choroidal and retinal damage, these prognostic suggestions are speculative and are not supported by outcome data in this manuscript. Formal validation of prognostic values requires dedicated prospective studies. Additionally, the current classification focuses on anatomical location and quadrant extent but does not differentiate between specific injury types (e.g., avulsion vs. laceration vs. tissue loss), which may have different surgical and prognostic implications. Future studies may refine the system by incorporating these variables. Moreover, interobserver reproducibility of the proposed zonal classification has not been assessed. Future prospective, multicenter studies should evaluate the consistency of zone assignment among different surgeons and its correlation with surgical decision‐making and outcomes. Future studies should be designed to evaluate whether the proposed uveal injury classification correlates with clinically meaningful endpoints, including anatomical success, visual outcomes, proliferative vitreoretinopathy, hypotony, and phthisis bulbi.

In summary, the proposal of uveal injury classification is a useful complement to existing classifications and zone system for mechanical ocular trauma. It can assess the extent of ocular damage and guides surgical treatment more accurately, thereby enhancing development of management and research in this field.

## Author Contributions

Tianjing Yang wrote the original manuscript and Hua Yan designed the study and revised the manuscript.

## Funding

This work was supported by grants from the National Natural Science Foundation of China (82530032, 82330031), the Natural Science Foundation of Tianjin (25JCZDJC00390), and the Tianjin Key Medical Discipline Construction Project (No. TJYXZDXK‐3‐004A).

## Disclosure

All authors read and approved the final manuscript.

## Ethics Statement

This study proposes a novel classification system for uveal injuries. The clinical data presented in the Methods section represent routine clinical experience from a single center and were used to inform the development of the classification. This was not a formal retrospective research study; therefore, upon review, the Ethics Committee of Tianjin Medical University General Hospital confirmed that formal ethical approval was not required. All patients provided informed consent for their data to be used for clinical and educational purposes.

## Consent

Please see the Ethics Statement.

## Conflicts of Interest

The authors declare no conflicts of interest.

## Supporting Information

Additional supporting information can be found online in the Supporting Information section.

## Supporting information


**Supporting Information** The supporting tables are available: Table S1 summarizes the demographic and clinical characteristics of the 108 patients with uveal injury; Table S2 compares the strengths and limitations of the proposed classification with existing systems.

## Data Availability

The datasets supporting the conclusions of this article are included within the article.

## References

[1] Yan H. , Yang K. , Ma Z. et al., Guideline for the Treatment of No Light Perception Eyes Induced by Mechanical Ocular Trauma, Journal of Evidence-Based Medicine. (2022) 15, no. 3, 302–314, 10.1111/jebm.12496. 36151612 PMC9826528

[2] Wang S. , Li F. , Jin S. , Zhang Y. , Yang N. , and Zhao J. , Biomechanics of Open-Globe Injury: A Review, Biomedical Engineering Online. (2023) 22, no. 1, 10.1186/s12938-023-01117-8. PMC10210437 37226242

[3] Chou B. W. , Feng S. , Ding L. , and Mudumbai R. C. , Lens Injury in Setting of Zone I and II Open Globe Injuries, Indian Journal of Ophthalmology. (2025) 73, no. 1, 59–63, 10.4103/ijo.Ijo_986_24. 39723855 PMC11831949

[4] Blanch R. J. , McMaster D. , and Patterson T. J. , Management of Open Globe Injury: A Narrative Review, Eye. (2024) 38, no. 16, 3047–3051, 10.1038/s41433-024-03246-3. 39085596 PMC11543839

[5] Victor A. A. , Violetta L. , Kusumowidagdo G. , and Pranata R. , Pars-Plana Vitrectomy Combined With Retinectomy in Severe Open-Globe Injuries: A Systematic Review and Meta-Analysis, European Journal of Ophthalmology. (2022) 32, no. 3, 1652–1661, 10.1177/11206721211029472. 34213376

[6] Dalma-Weiszhausz J. and Dalma A. , The Uvea in Ocular Trauma, Ophthalmology, An Issue of Medical Clinics of North America. (2002) 15, no. 2, 205–213, 10.1016/s0896-1549(02)00016-0. 12229237

[7] Pelletier J. , Koyfman A. , and Long B. , High Risk and Low Prevalence Diseases: Open Globe Injury, The American Journal of Emergency Medicine. (2023) 64, 113–120, 10.1016/j.ajem.2022.11.036. 36516669

[8] Kuhn F. , Morris R. , Witherspoon C. D. , Heimann K. , Jeffers J. B. , and Treister G. , A Standardized Classification of Ocular Trauma, Ophthalmology. (1996) 103, no. 2, 240–243, 10.1016/s0161-6420(96)30710-0. 8594508

[9] Pieramici D. J. , Sternberg P. , Aaberg T. M. et al., A System for Classifying Mechanical Injuries of the Eye (Globe). the Ocular Trauma Classification Group, American Journal of Ophthalmology. (1997) 123, no. 6, 820–831, 10.1016/s0002-9394(14)71132-8. 9535627

[10] Feng K. , Hu Y. T. , and Ma Z. , Prognostic Indicators for No Light Perception After Open-Globe Injury: Eye Injury Vitrectomy Study, American Journal of Ophthalmology. (2011) 152, no. 4, 654–62.e2, 10.1016/j.ajo.2011.04.004. 21726850

[11] Puodžiuvienė E. , Valeišaitė G. , and Žemaitienė R. , Clinical Characteristics, Visual Outcomes, and Prognostic Factors of Open Globe Injuries, Medicina. (2021) 57, no. 11, 10.3390/medicina57111198. PMC8618771 34833416

[12] Andreoli M. T. and Andreoli C. M. , Surgical Rehabilitation of the Open Globe Injury Patient, American Journal of Ophthalmology. (2012) 153, no. 5, 856–860, 10.1016/j.ajo.2011.10.013. 22265150

[13] Ung C. , Stryjewski T. P. , and Eliott D. , Indications, Findings, and Outcomes of Pars Plana Vitrectomy After Open Globe Injury, Ophthalmol Retina. (2020) 4, no. 2, 216–223, 10.1016/j.oret.2019.09.003. 31732470

[14] Iwase T. , Nishiyama S. , and Sato M. , A Novel Suturing Technique for Choroidal Avulsion, Journal of Clinical Medicine. (2022) 11, no. 18, 10.3390/jcm11185344. PMC9504976 36142991

[15] Ma J. , Tong Y. , Shen Z. , and Ye P. , Efficacy of Combined Vitreous Surgery and Choroidal Suture Fixation on Choroidal Avulsion, Eye Science. (2011) 26, no. 3, 143–147, 10.3969/j.issn.1000-4432.2011.03.004. 21913345

[16] Mittra R. A. and Mieler W. F. , Controversies in the Management of Open-Globe Injuries Involving the Posterior Segment, Survey of Ophthalmology. (1999) 44, no. 3, 215–225, 10.1016/s0039-6257(99)00104-6. 10588440

[17] Jin Y. , Chen H. , Xu X. , Hu Y. , Wang C. , and Ma Z. , Traumatic Proliferative Vitreoretinopathy: Clinical and Histopathological Observations, Retina. (2017) 37, no. 7, 1236–1245, 10.1097/iae.0000000000001350. 27779559

[18] Chauhan M. Z. , Georgiou M. , Al-Hindi H. , and Uwaydat S. H. , Outcomes of Pars Plana Vitrectomy Following Ocular Trauma at Varying Surgical Time Points, International Journal of Retina and Vitreous. (2022) 8, no. 1, 10.1186/s40942-022-00399-9. PMC9310478 35879788

[19] Kuhn F. and Morris R. , Early Vitrectomy for Severe Eye Injuries, Eye. (2021) 35, no. 5, 1288–1289, 10.1038/s41433-020-01308-w. 33235339 PMC8182830

[20] Yan H. , Chen W. , Zhao Z. et al., Expert Consensus on the Clinical Application of Artificial Iris, Journal of Evidence-Based Medicine. (2026) 19, no. 1, 10.1111/jebm.70101. 41485122

