# Supplementary material for: A Standardized Classification of Uveal Injury
Source: J Ophthalmol. 2026 Jul 13;2026:8878849. doi: 10.1155/joph/8878849 (PMC13358804; doi:10.1155/joph/8878849)
Supplement: Supplementary file 1 — Supporting Information The supporting tables are available: Table S1 summarizes the demographic and clinical characteristics of the 108 patients with uveal injury; Table S2 compares the strengths and limitations of the proposed classification with existing systems. [file JOPH-2026-8878849-s001.zip › Table S1.docx]

Table S2. Demographic and clinical characteristics of patients with uveal injury

| **Item** | **Value** |
| --- | --- |
| **Demographics** |  |
| **Number of patients (eyes)** | 108 (110) |
| **Age (years), mean ± SD (range)** | 41.2 ± 14.6 (6–79) |
| **Sex, n (%)** |  |
| Male | 72 (66.7) |
| Female | 36 (33.3) |
| **Inclusion criteria** | Mechanical ocular injury patients with uveal involvement requiring surgical intervention |
| **Injury mechanism, n (%)** |  |
| Blunt contusion | 34 (30.9) |
| Penetrating injury | 28 (25.5) |
| Perforating injury | 8 (7.3) |
| Globe rupture | 23 (20.9) |
| Intraocular foreign body | 17 (15.5) |
| **Follow‑up duration, median (range), months** | 4 (0.5–24) |
| **Anatomical reposition rate, %** |  |
| Zone I | 100 |
| Zone II | 100 |
| Zone III | 83.3 |
| Zone IV | 92.9 |
| **Complications, n (%)** |  |
| Proliferative vitreoretinopathy | 18 (16.4) |
| Hypotony | 8 (7.3) |
| Endophthalmitis | 1 (0.9) |
| Recurrent retinal detachment | 3 (2.7) |
