# Supplementary material for: A Standardized Classification of Uveal Injury
Source: J Ophthalmol. 2026 Jul 13;2026:8878849. doi: 10.1155/joph/8878849 (PMC13358804; doi:10.1155/joph/8878849)
Supplement: Supplementary file 1 — Supporting Information The supporting tables are available: Table S1 summarizes the demographic and clinical characteristics of the 108 patients with uveal injury; Table S2 compares the strengths and limitations of the proposed classification with existing systems. [file JOPH-2026-8878849-s001.zip › Table S2.docx]

| **System** | **Strengths** | **Limitations** |
| --- | --- | --- |
| Kuhn classification (1996) | Classifies ocular trauma based on injury mechanism (open globe, closed globe, etc.) | Does not classify by anatomical location of posterior ocular tissue injury |
| Pieramici zone system (1997) | Classifies by anatomical location of globe wall injury (Zones I, II, III) | Does not classify uveal injury by anatomical location |
| Proposed uveal classification | Classifies uveal injury by anatomical location (Zones I–IV by most posterior involvement) | Does not classify all ocular tissue injuries |

Table S1. Comparison of the strengths and limitations of each classification system
